# Molecular Mechanisms of Bipolar Disorder: Progress Made and Future Challenges

**DOI:** 10.3389/fncel.2017.00030

**Published:** 2017-02-14

**Authors:** Yeni Kim, Renata Santos, Fred H. Gage, Maria C. Marchetto

**Affiliations:** ^1^Laboratory of Genetics, The Salk Institute for Biological StudiesLa Jolla, CA, USA; ^2^Department of Child and Adolescent Psychiatry, National Center for Mental HealthSeoul, South Korea; ^3^Ecole Normale Supérieure, PSL Research University, Centre National de la Recherche Scientifique (CNRS), Institut National de la Santé et de la Recherche Médicale (INSERM), Institut de Biologie de l’Ecole Normale Supérieure (IBENS)Paris, France

**Keywords:** bipolar disorder, mitochondrial dysfunction, endoplasmic reticulum stress, oxidative stress, glutamate, hyperexcitability, disease modeling

## Abstract

Bipolar disorder (BD) is a chronic and progressive psychiatric illness characterized by mood oscillations, with episodes of mania and depression. The impact of BD on patients can be devastating, with up to 15% of patients committing suicide. This disorder is associated with psychiatric and medical comorbidities and patients with a high risk of drug abuse, metabolic and endocrine disorders and vascular disease. Current knowledge of the pathophysiology and molecular mechanisms causing BD is still modest. With no clear biological markers available, early diagnosis is a great challenge to clinicians without previous knowledge of the longitudinal progress of illness. Moreover, despite recommendations from evidence-based guidelines, polypharmacy is still common in clinical treatment of BD, reflecting the gap between research and clinical practice. A major challenge in BD is the development of effective drugs with low toxicity for the patients. In this review article, we focus on the progress made and future challenges we face in determining the pathophysiology and molecular pathways involved in BD, such as circadian and metabolic perturbations, mitochondrial and endoplasmic reticulum (ER) dysfunction, autophagy and glutamatergic neurotransmission; which may lead to the development of new drugs.

## Introduction

In his 1889 lecture “Zur Diagnose und Prognose der Dementia praecox”, Emil Kraepelin proposed separating psychiatric disorders with psychotic features into two major categories. Based on the observations of the long-term outcome and the nosological principles of Kahlbaum (1863), the famous diagnostic dichotomy was born: the “Manisch-depressives Irresein” which was later reclassified to bipolar disorder (BD) and major depression, and the “Dementia-praecox-Gruppe” which became schizophrenia. BD is a complex syndrome with 2% prevalence worldwide (Merikangas et al., 2011). The impact of BD on patients can be devastating; 9%–15% of patients commit suicide (Rihmer and Kiss, 2002; Medici et al., 2015). This disorder is associated with psychiatric comorbidities including personality disorder, anxiety disorder and substance abuse disorder and medical comorbidities such as diabetes, obesity and hyperlipidemia (Leboyer et al., 2012; Blanco et al., 2017).

Even with typical symptoms of BD, the disease is difficult to diagnose accurately and promptly in clinical practice. Both BD types I and II patients spend most of the duration of their illness in a depressive phase (Hirschfeld et al., 2003) and they often fail to recognize hypomanic or manic symptoms as pathological, which results in a mean delay of 5–10 years between the onset of illness and diagnosis (Baldessarini et al., 2007). There are also subtle differences between the two major diagnostic criteria used throughout the world today, the DSM and the International Classification of Disease (ICD). According to DSM-5 criteria, BD type I is diagnosed when there has been at least one episode of full-blown mania, with or without one or more major depressive or hypomanic episodes (American Psychiatric Association, 2013). A diagnosis of BD II is based on several protracted episodes and at least one hypomanic episode but no manic episodes. The ICD-10 does not discriminate between BD types I and II (WHO, 1993). While one episode of mania or one episode of hypomania plus major depressive episodes is sufficient for diagnosis according to DSM-5, the ICD-10 requires at least two distinct mood episodes, one of which must be manic or hypomanic for the diagnosis of BD. With no clear clinically relevant biological markers available, early diagnosis is a great challenge for clinicians without knowledge of the longitudinal progress of illness (Phillips and Kupfer, 2013).

Treatment of BD usually consists of two stages: acute stabilization and relapse prevention. Acute stabilization entails the conversion of a manic or depressive phase to an euthymic state; relapse prevention consists of maintaining the euthymic status while minimizing subthreshold symptoms and enhancing general function (Geddes and Miklowitz, 2013; Goodwin et al., 2016). Acute treatment of BD is complex, as one mood phase may spill over into the opposite mood phase before the euthymic status can be achieved, complicating the various aspects of clinical decisions e.g., the choice of psychotropics and the dosage. Also teasing out the therapeutic effects from the possible adverse effects such as somnolence, psychomotor retardation and akathisia, all of which may mimic a change in the mood status, is one of the biggest challenges during the initial phase of BD drug treatment. A meta analysis of short-term randomized control trials of medications showed that aripiprazole, asenapine, carbamazepine, cariprazine, haloperidol, lithium, olanzapine, paliperdone, quetiapine, risperidone, tamoxifen, valproate and ziprasidone were effective as acute anti-manic agents (Yildiz et al., 2011). The same study also showed that responses to various antipsychotics were somewhat greater or more rapid than lithium, valproate, or carbamazepine and that lithium did not differ from valproate in a direct comparison between the drugs (Yildiz et al., 2011). Relapse prevention usually necessitates long-term treatment that calls for drugs that have minimal long-term side effects. Lithium is the best-established long-term treatment compound for BD, reducing both relapse and suicide (Geddes et al., 2004; Nivoli et al., 2010; Rybakowski, 2014). However, the incidence of adverse effects and a low therapeutic index restrict its benefits. Despite recommendations from evidence-based guidelines, polypharmacy is still common in clinical practice of BD, reflecting the gap between research and routine clinical practice (Fornaro et al., 2016).

The reduced understanding of the underlying pathophysiology and neurobiology of the disorder hampered the development of effective drugs. Neuroimaging studies have consistently revealed structural changes in the brain of BD patients (Maletic and Raison, 2014). In addition, observation of post-mortem tissue showed histopathologic features in neurons and glia in BD (Rajkowska, 2000; Uranova et al., 2004). These findings encouraged moving the research from monoamine neurotransmission to the synaptic and neural plasticity and the cellular processes that control the physiology of brain cells. In this review article, we will focus on how alterations in the energetic metabolism and mitochondrial dysfunction contribute to the vulnerability of BD cells, which we expect may lead to future therapies.

## Circadian and Metabolic Perturbations in BD

**Figure 1:**
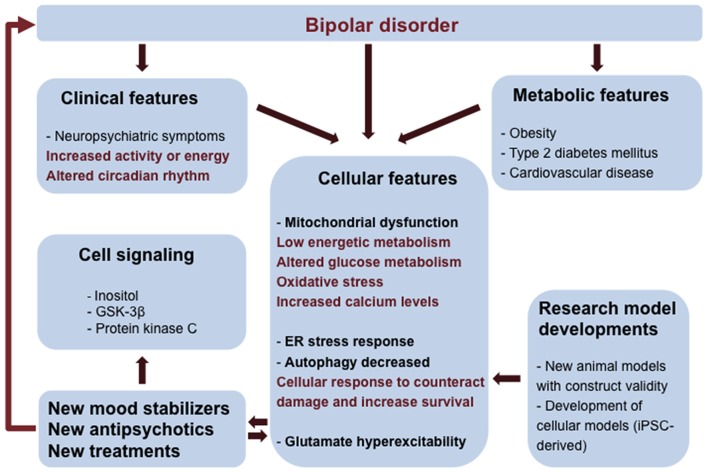
**Integrated view of clinical and fundamental research interventions in bipolar disorder (BD).** BD patients have neuropsychiatric symptoms and metabolic comorbidities that can be associated to mitochondrial dysfunction and low energetic status. Oxidative stress, endoplasmic reticulum (ER) stress, reduced autophagy and changes in glutamatergic neurotransmission are consequences of mitochondrial dysfunction and altered glucose metabolism contributing to the vulnerability of BD cells. Clinical and cellular features can be used to inform and validate cellular phenotypes useful in the construction of new research model systems (mouse models and induced pluripotent stem cells- iPSCs technology). Elucidation of pathways involved in BD pathology can lead to the development of novel therapies.

The clinical manifestations and the pathogenesis of BD are linked to circadian rhythm alteration (Melo et al., 2016a,b; Figure 1). Circadian disruptions and sleep complaints can be both precipitating factors and consequences of mood disorders (Bechtel, 2015; Cretu et al., 2016; Grierson et al., 2016). One of the main characteristics of manic episodes is the reduced need for sleep, whereas depressive episodes are frequently characterized by insomnia and hypersomnia (American Psychiatric Association, 2013). Circadian disruption and “eveningness” (being more active during the evening) have been associated with mood episodes, functional impairment, poor quality of life and treatment resistance (Duarte Faria et al., 2015; Pinho et al., 2015; Cudney et al., 2016; Ng et al., 2016). Moreover, sleep deprivation and light therapy are therapeutic approaches that have been used effectively as adjuncts to the more standard pharmacological therapies (Lewy et al., 1982; Benedetti et al., 2014; Tseng et al., 2016).

Existing hypotheses for the biological mechanism underlying dysregulation of circadian rhythm in BD include changes in melatonin levels, in expression of melatonin receptors in the central nervous system and in daily cortisol profiles (Wu et al., 2013). Genetic evidence also links circadian rhythm dysregulation with BD. Two polymorphisms on the CLOCK gene that control circadian rhythm—aryl hydrocarbon receptor nuclear translocator-like (ARNTL) and timeless circadian clock (TIMELESS)—have been linked to lithium responsiveness in BD (Rybakowski et al., 2014). In addition, Per2, Cry1 and Rev-Erbα expression, all components of the circadian clock, increased the individual susceptibility to the therapeutic effects of lithium (Schnell et al., 2015).

It is interesting to note that circadian rhythm dysregulation and molecular clock mechanism are observed across psychiatric diagnoses, including schizophrenia and depression (Lamont et al., 2007). In a genome-wide association analysis of UK biobank, genetic correlation between longer sleep duration and schizophrenia risk was observed (Lane et al., 2017). In addition, a SNP analysis showed *CLOCK* gene T3111C polymorphism in Japanese schizophrenia patients compared to healthy controls (Takao et al., 2007), although the same polymorphism was not observed in patients with major depressive disorder (MDD) or BD in another study of the Japanese population (Kishi et al., 2011). Recently, it was observed in primary fibroblasts from schizophrenic patients with poor sleep, a loss of rhythmic expression of CRY1 and PER2 when compared to healthy controls (Johansson et al., 2016). Increased sleep latency, poor sleep quality and reduced latency to first rapid eye movement sleep are well documented in MDD, but there is no data supporting the role of circadian rhythm genes in the disorder (Thase, 2006).

Metabolic cues contribute to the regulation of circadian clocks and circadian rhythm impact the cardiovascular and metabolic systems (Morris et al., 2012; Gamble and Young, 2013; Bailey et al., 2014). Indeed it has long been known that BD patients present energetic metabolism changes (Altschule et al., 1956; Kato and Kato, 2000) and that they have a higher risk of obesity (Boudebesse et al., 2015) and type 2 diabetes mellitus compared to the general population (McElroy et al., 2002; Keck and McElroy, 2003; McIntyre et al., 2005). Systemic analysis have shown that natural causes like cardiovascular illness contribute significantly to the decrease in the life expectancy of BD patients compared to the general population (Kessing et al., 2015). Circadian disturbance appears to be independently associated with increased lipid peroxidation in BD patients but not in controls (Cudney et al., 2014). An association between evening chronotype and a higher percentage of body fat composition among patients with BD has been suggested (Soreca et al., 2009). Many cellular metabolic sensors act directly on core components of the clock, adjusting biological timing with metabolic status. Leptin, which is produced by adipocytes, regulates appetite and modulates sleep duration. Increased levels of leptin have been described previously in overweight patients with BD as compared with overweight controls (Barbosa et al., 2012). Adipose tissue-derived hormones, or adipokines, regulate appetite and metabolism and have activity in limbic brain regions; mood episodes and medication treatment both contribute to adipokine abnormalities in BD and adipokines influence the course of psychiatric illness and changes in BMI (Bond et al., 2016).

The mechanisms underlying weight gain and metabolic imbalance in BD patients are poorly understood. Genetic susceptibility, recurrent depressive episodes, low activity levels, poor dietary habits, poor medical care, and side effects of antipsychotics/mood-stabilizers medication have been suggested (McElroy et al., 2002). Sleep disturbance is a core symptom of BD and may contribute to the association of BD with metabolic disturbances. Evidence indicates that shorter sleep duration is associated with low HDL cholesterol (Soreca et al., 2012) and increased risk of coronary events (Ayas et al., 2003). However, other studies suggest that comorbid medical illnesses of BD may not only be due to poor health behaviors and psychotropic medications, but manifestations of common biological pathways between the BD and the comorbid illnesses (Leboyer et al., 2012). The close associations between metabolic and psychiatric disorders have introduced the “metabolic mood syndrome” hypothesis, which speculates the existence of common biological mechanisms underlying both conditions.

The innate energetic glucose-dependent brain metabolism may be one of the factors that contribute to this phenomena. Our brain has a very high-energy requirement and will disturb other parts of the body to acquire its energetic need, which is the core of the “selfish brain” hypothesis (Peters et al., 2004). We can hypothesize that some of the metabolic changes observed in BD may be a compensatory mechanism of the body trying to offset the pathological energy imbalance during the initial stages of BD characterized by loss of appetite, increased energy and lack of sleep, all of which would deplete the body and brain of energy sources. Maybe it is not a coincidence that many of the initial side effects of antipsychotics and mood stabilizers (appetite increase and somnolence) used to treat manic episodes, point toward the body trying to tip the scale toward anabolism instead of catabolism. The previous findings have suggested that high BMI impacts negatively on clinical and functional outcomes in BD (Kolotkin et al., 2008), adversely influencing treatment response to mood stabilizers and remission rate (Kemp et al., 2010). However, higher weight gain may be a compensatory response to more severe pathological process than the cause of the negative clinical outcome and poor drug response. Although a shared risk and overlapping pathophysiology implicate either shared biological mechanisms or causal interactions for circadian rhythm, metabolic disturbances and BD, more research is needed to study the specific mechanisms in place.

## Mitochondrial Dysfunction and Energy Metabolism

Accumulating evidence from imaging, biochemical and genetic studies support the view that mitochondrial dysfunction is a central feature in BD (Kato and Kato, 2000), characterized by impaired oxidative phosphorylation and changes in mitochondrial morphology and number and in calcium signaling (Figure 1). Several mitochondrial DNA polymorphisms have been described (Kato et al., 2001; Munakata et al., 2004), providing additional support for the association between BD and mitochondrial impairment.

**Figure 2:**
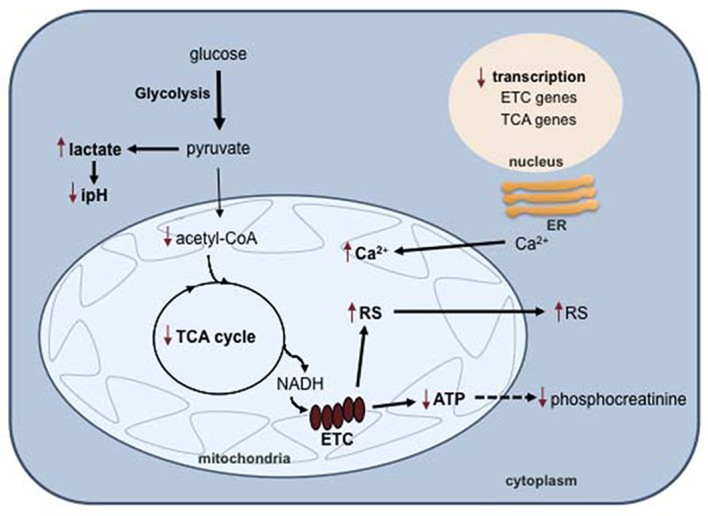
**Mitochondrial dysfunction in BD.** In BD cells, impaired oxidative phosphorylation results in a metabolic switch to glycolysis and lactate biosynthesis, with concomitant intracellular pH decrease. Decreased oxidative phosphorylation also causes an accumulation of reactive species (RS) and calcium in the mitochondria. Abbreviations: ER, endoplasmic reticulum; ETC, electron transport chain; TCA, tricarboxylic acid cycle; RS, reactive species.

Magnetic resonance spectroscopy (MRS) studies were the first to show perturbations in several pathways involved in energy metabolism in BD patients. In a pioneering study using phosphorous ^31^P-MRS, Kato et al. (1994) identified reduced phosphocreatinine in the frontal cortex of BD patients regardless of mood phase; this finding was later confirmed by other studies using a different cohort (Deicken et al., 1995; Frey et al., 2007). Phosphocreatinine is a cellular reservoir for ATP synthesis in periods of intense metabolic demand, and a chronic decrease in phosphocreatinine levels is an indication of mitochondrial dysfunction and deficient ATP synthesis. Inorganic phosphate regulates oxidative phosphorylation and ATP synthesis (Brown, 1992; Bose et al., 2003). Based on early pioneering studies, Stork and Renshaw (2005) proposed a model of mitochondrial dysfunction in which a metabolic shift towards glycolysis occurs in the brain of BD individuals (Figure 2). A recent ^31^P-MRS studies in adolescents showed that inorganic phosphate was decreased in medication-free patients compared to medicated patients and controls (Shi et al., 2012), again emphasizing the low energy status of BD brain cells.

The tricarboxylic acid cycle (TCA) is a fundamental component of aerobic respiration and is also disturbed in BD (Figure 2). Metabolomic analysis showed that the serum levels of pyruvate and α-ketoglutarate were significantly higher in BD patients than in healthy controls (Yoshimi et al., 2016b). Pyruvate is an end product of glycolysis and is used to fuel the TCA cycle in the mitochondria after conversion into acetyl-CoA. α-Ketoglutarate is a TCA intermediate that results from the oxidative decarboxylation of isocitrate catalyzed by isocitrate dehydrogenase. The levels of this enzyme were found to be significantly higher in the cerebrospinal fluid (CSF) of BD patients compared to neurotypical controls, possibly accounting for the α-ketoglutarate increase (Yoshimi et al., 2016a). Studies using proton ^1^H-MRS detected decreased intracellular pH and increased lactate in several brain regions of BD patients compared to healthy individuals (Kato et al., 1994; Dager et al., 2004; Chu et al., 2013). Accordingly, the lactate level in the CSF is higher in BD patients compared to healthy controls (Regenold et al., 2009). At the transcriptional level, different studies reported post-mortem decreased expression of genes encoding numerous subunits of complexes I, III, IV and V of the electron transport chain in the hippocampus (Konradi et al., 2004) and prefrontal cortex (Iwamoto et al., 2005; Sun et al., 2006; Andreazza et al., 2010) of BD patients. All of these observations converge to support the hypothesis that a metabolic shift occurs from oxidative phosphorylation to the less-efficient pathway glycolysis in the brain of BD individuals (Figure 2).

Mitochondria accomplish other cellular functions, such as the regulation of calcium homeostasis and of cell death (McBride et al., 2006; Giacomello et al., 2007; Suen et al., 2008; Bhosale et al., 2015). At the same time, mitochondria actively participate in the intracellular regulation of calcium signaling by buffering the calcium waves. Lethal challenges stimulate calcium release by the endoplasmic reticulum (ER) and uptake by mitochondria, which are early steps in the apoptotic cascade, and the capacity of mitochondria to handle calcium fluxes will determine survival or death (Giacomello et al., 2007; Bhosale et al., 2015; Raffaello et al., 2016). In general, an intensification in cellular energy demand is associated with increased calcium (Bhosale et al., 2015). Since BD patient cells have impaired oxidative phosphorylation, it is likely that they also have disturbed calcium homeostasis. However, only a few studies have addressed these aspects of mitochondrial function in the context of BD. As expected, markers of apoptosis and an increase in the intracellular calcium concentration were found in blood cells from BD patients compared to healthy individuals (Perova et al., 2008; Dubovsky et al., 2014; Fries et al., 2014). Underscoring the potential role for calcium homeostasis in BD pathogenesis is the repeated identification of CACNA1C, which encodes the α-subunit of the L-type voltage-gated Ca^2+^ channel, as a risk gene (Maletic and Raison, 2014).

Mitochondria undergo continuous fusion and fission events in physiological conditions. The imbalance of these two processes has dramatic effects on the morphology, physiology and distribution of mitochondria in the cells (Detmer and Chan, 2007; Ramos et al., 2016; Schrepfer and Scorrano, 2016). When the equilibrium is directed towards fusion, mitochondria are interconnected, net-like or aggregated in small regions of the cell. When the equilibrium is directed towards fission, mitochondria are fragmented, respiration-incompetent and tend to lose mitochondrial DNA. The data from Cataldo et al. (2010) suggest alterations in mitochondrial morphology, number and distribution in post mortem prefrontal cortex samples and primary fibroblasts and lymphocytes from BD individuals compared to controls. The mitochondria were also smaller in neurons differentiated from induced pluripotent stem cells (iPSC) from BD patients compared to healthy controls (Mertens et al., 2015). Considering that BD is characterized by low energy status, it is tempting to speculate that these studies are reporting altered mitochondrial dynamics. In both studies, treatment with lithium did not cause any change in mitochondria (Cataldo et al., 2010; Mertens et al., 2015). There is no treatment strategy targeting metabolism in BD, most studies aim at finding biomarkers or at better understanding the pathology. However, drugs commonly used for treatment of metabolic disease may have beneficial effects on BD metabolism; for example, quetiapine reduces lactate (Kim et al., 2007) and lithium increases oxidative phosphorylation (Maurer et al., 2009).

## BD and Oxidative Stress

One outcome of oxidative phosphorylation decline is the increase in the generation of superoxide as a result of electron leak from the electron transport chain, which may lead to oxidative stress. A cell is in an oxidative stress state when an imbalance between the production of reactive species (RS) and antioxidant activities occurs (Halliwell, 2007). Increasing evidence suggests the involvement of oxidative stress in the pathology and progression of BD (Scaini et al., 2016; Data-Franco et al., 2017).

Two meta-analysis studies have shown that lipid peroxidation and nitric oxide level were significantly increased in red blood cells or serum from BD patients compared to healthy controls (Andreazza et al., 2008a; Brown et al., 2014). Oxidative damage of nucleic acids was also repeatedly observed and was found to be increased in peripheral and post-mortem patient brain samples (Andreazza et al., 2008a; Che et al., 2010; Soeiro-de-Souza et al., 2013; Brown et al., 2014). However, numerous studies have reported contradictory data on the antioxidant enzymatic activities (e.g., superoxide dismutase, catalase, glutathione peroxidase) in BD patients (Brown et al., 2014). In addition, using ^1^H-MRS, no change was observed in the levels of glutathione, a major antioxidant in the brain, in the anterior cingulate cortex of BD patients and healthy controls (Chitty et al., 2013; Lagopoulos et al., 2013). On the other hand, a biochemical study of blood samples from patients with different ages of disease onset showed that glutathione levels are lower in BD patients and that a negative correlation was observed with the age at onset (Rosa et al., 2014). The discrepancies reported could be related to the heterogeneity of the studies in terms of type of tissue analyzed, age at onset, illness duration, phase of the disorder, number of manic/depression episodes and treatment. The cellular effects of oxidative stress are cumulative and it is predictable to worsen with time and number of manic episodes. Hatch et al. (2015) observed that protein carbonyl and lipid hydroperoxide content is higher in adults compared to adolescents with BD. Another study showed that, indeed, antioxidant defenses might oscillate according to the phase of the disorder; superoxide dismutase activity was higher in manic and depressed patients compared to euthymic patients and controls (Andreazza et al., 2007). Notably, numerous reports have shown the antioxidant properties of mood stabilizers (Cui et al., 2007; Andreazza et al., 2008b; Bakare et al., 2009; Jornada et al., 2011; Banerjee et al., 2012; de Sousa et al., 2014).

The interest of researchers on the effects of oxidative stress on the pathophysiology of BD is recent; therefore the available data is limited. Over the years a number of trials with antioxidants have failed to provide the expected benefits for patients with various diseases (Casetta et al., 2005; Steinhubl, 2008; Halliwell, 2009; Persson et al., 2014). One of the reasons is because oxidative stress is frequently a secondary phenotype of mitochondrial dysfunction, as it is likely the case in BD patients. Further research is needed to evaluate the therapeutic potential of antioxidants and it’s efficacy when given as adjunctive treatments.

## Link Between Mitochondrial Dysfunction, Endoplasmic Reticulum Stress and Autophagy

**Figure 3:**
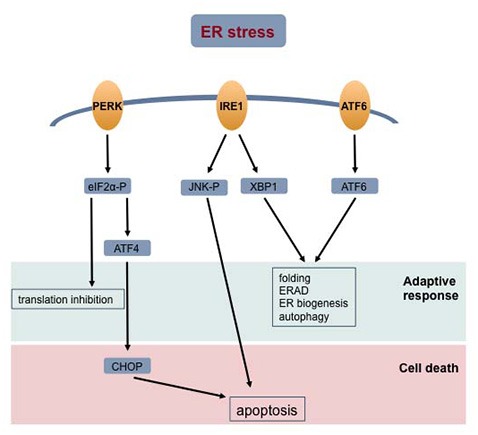
**Cellular response to ER stress.** Accumulation of unfolded proteins in the ER lumen signal the unfolded protein response (UPR). The activated stress sensor proteins—protein kinase RNA-like ER kinase (PERK), inositol-requiring enzyme 1 (IRE1) and activating transcription factor 6 (ATF6) –signal different transduction pathways aiming at restoring cell homeostasis or committing the cell to death. Abbreviations: ERAD, endoplasmic reticulum-associated protein degradation; ER, endoplasmic reticulum.

The principal functions of the ER are protein synthesis, folding and post-translational modifications, but it also interacts functionally with mitochondria to control calcium signaling and apoptosis (Pizzo and Pozzan, 2007; Halperin et al., 2014; Senft and Ronai, 2015; Raffaello et al., 2016). Accumulation of unfolded proteins, which may be triggered by defaults in protein folding or post-translational modifications, calcium changes and by redox imbalance, causes ER stress. The cellular response to ER stress involves other organelles, such as the mitochondria, which leads to restoring cell homeostasis or to committing cells to death. The pathways activated by ER stress are the unfolded protein response (UPR), ER-associated degradation, autophagy, hypoxic signaling and mitochondrial biogenesis (Raffaello et al., 2016). The UPR is mediated by three stress sensors—the inositol-requiring enzyme 1 (IRE1), the activating transcription factor 6 (ATF6) and protein kinase RNA-like ER kinase (PERK)—that activate a complex transcriptional cascade that leads to multiple adaptive responses or cell death (Hetz, 2012; Senft and Ronai, 2015; Figure 3).

Several studies using BD patient-derived lymphoblastoid cell lines or blood cells showed an impaired response to ER stress (So et al., 2007; Hayashi et al., 2009; Pfaffenseller et al., 2014). So et al. (2007) were the first to report decreased induction of expression of the X-box binding protein 1 (XBP1) and C/EBP homologous protein (CHOP) genes in response to ER stress stimulated by thapsigargin and tunicamycin in B-lymphocytes from BD patients compared to controls. In agreement with these observations, another study showed an increase in the amount of several proteins implicated in the UPR (phosphorylated eukaryotic initiation factor 2 (eIF2α-P), chaperone GRP78, XBP1 and CHOP) in leucocytes treated with tunicamycin from controls but not in those from BD patients (Pfaffenseller et al., 2014). Hayashi et al. (2009) also reported an attenuation in the expression of XBP1 and GRP94 in BD patient-derived lymphoblastoid cell lines treated with thapsigargin compared to the control cell lines. A single nucleotide polymorphism (SNP; −116C/G; rs2269577) in the promoter of the XBP1 gene impairs the feedback loop regulation in the ER response and is associated with BD (Kakiuchi et al., 2003; Cheng et al., 2014). However, the attenuated XBP1 induction in patient B-lymphocytes was independent of the promoter polymorphism (So et al., 2007). Differential gene analysis of data obtained by RNA sequencing from blood cells from healthy controls, lithium-responsive patients and lithium-non-responsive patients identified the response to ER stress as a lithium-regulated gene network (Breen et al., 2016). Overall, these data suggest that the adaptive response of BD cells to ER stress is compromised, which may decrease the resilience of cells to stress conditions (Figure 3). Interestingly, the most frequently used mood stabilizers, lithium and valproate, seem to also have beneficial effects and increase the adaptive response to ER stress.

Autophagy is a cellular response aiming at restoring homeostasis or committing cells to death under nutrient starvation conditions (Klionsky and Emr, 2000; Baehrecke, 2005). During autophagy, protein aggregates, cytoplasmic components and organelles are degraded and the released molecules are recycled in biosynthetic pathways. The molecular mechanisms that regulate autophagy are the activation of ATG (autophagy-related) genes by phosphatidylinoditol 3-kinase (PI3K) pathway and the repression of the mTOR (mammalian target of rapamycin) kinase (Klionsky and Emr, 2000). A complex interaction exists between autophagy, ER stress and mitochondria. For instance, the UPR mediators activate autophagy (Figure 3; Senft and Ronai, 2015; Raffaello et al., 2016) and affect mitochondrial function by regulating Parkin (Bouman et al., 2011). On the other hand, Parkin is a regulator of mitochondrial dynamics and is necessary to target damaged mitochondria to mitophagy (Narenda et al., 2008; Poole et al., 2008). Recently, it was shown that autophagy is down regulated in schizophrenia and MDD (Jia and Le, 2015; Merenlender-Wagner et al., 2015). No data is available hitherto for patient cells or animal models of BD. However, since there is evidence that mitochondrial and ER functions are disturbed in BD, it is possible that autophagy is also altered and contributes to the pathophysiology of the disorder. Toker and Agam (2015) suggested an original hypothesis; they proposed that in psychiatric disorders mitochondrial dysfunction results from autophagy impairment.

## Glutamatergic Neurotransmission and Hyperexcitability

Glutamate and γ-aminobutyric acid (GABA) are the major excitatory and inhibitory neurotransmitters in the brain, respectively. The most abundant neurons in the cortex are the excitatory pyramidal cells, the inhibitory interneurons account for only ≈20% of the total neurons (Markram et al., 2004; Molyneaux et al., 2007). A chronic accumulation of glutamate in the synaptic cleft causes excitotoxicity and neuronal death due to excessive stimulation of the postsynaptic glutamate receptors (Wang and Qin, 2010). A metabolic glutamate-glutamine cycle between neurons and astrocytes maintain glutamate levels below toxicity. Neuronal impulses trigger the release of glutamate into the synaptic cleft, generating postsynaptic currents. Glutamate is then taken up by astrocytes and converted into the non-toxic glutamine, which is transported back by the neurons and converted to glutamate. It is thus predictable that changes in the astrocyte ability to transport glutamate and synthesize glutamine will affect neurotransmission and neuronal survival in the brain.

The question of brain glutamate levels in BD patients has been addressed in numerous studies using MRS. The magnetic field strengths and signal-to-noise ratio in most studies using human individuals do not allow a fine resolution of the peak into glutamate and glutamine, so it is a composite peak of two metabolites that is quantified (generally named Glx; Stork and Renshaw, 2005). Since glutamate is in large supply compared to glutamine, it is assumed that the changes observed in the Glx signal are correlated with glutamate (Stork and Renshaw, 2005; Gigante et al., 2012). Adult BD patients show a consistent increase in glutamate levels in the frontal brain areas compared to healthy controls; these increases are independent of the mood phase (Castillo et al., 2000; Michael et al., 2003; Dager et al., 2004; Yildiz-Yesiloglu and Ankerst, 2006; Hashimoto et al., 2007; Moore et al., 2007; Eastwood and Harrison, 2010; Gigante et al., 2012; Kondo et al., 2014; Ehrlich et al., 2015). Treatment of patients with the multi-target mood stabilizers lithium and valproate restores the Glx levels to normal (Friedman et al., 2004; Strawn et al., 2012). Findings in post-mortem brain tissue from BD patients confirmed changes in glutamatergic neurotransmission. Hashimoto et al. (2007) reported an increase in the levels of glutamate in BD patient samples from frontal cortices. Proteomics and transcription studies showed alterations in N-methyl-D-aspartate (NMDA) receptors and other intermediates of glutamatergic signaling (Hashimoto et al., 2007; Rao et al., 2009; Eastwood and Harrison, 2010; Gottschalk et al., 2015). Accordingly, the SLC1A2 gene that encodes the astrocytic excitatory amino acid transporter 2 (EAAT2, responsible for majority of glutamate re-uptake in the brain) is a susceptibility locus to BD (Fiorentino et al., 2014).

A relationship was found between glucose metabolism and glutamatergic neuronal function *in vivo* in the rat cortex by measuring the rates of TCA cycle and glutamate synthesis using ^14^C-MRS (Sibson et al., 1998). A stoichiometry close to 1:1 was calculated between glucose metabolism and glutamate cycling, suggesting that the majority of the glucose consumed and energy produced in the cortex supports the glutamatergic synaptic activity (Sibson et al., 1998). In inhibitory neurons, GABAergic transmission also imposes high energy expense (Patel et al., 2005). Therefore, the increased levels of excitatory neurotransmitter glutamate in the brain of BD patients imply a higher energy demand on the neurons. Dager et al. (2004) suggested that the increased rate of glycolysis observed in MRS studies is the metabolic response of BD cells to the increased energy requirements and to the deficient oxidative metabolism. Consistent with increased glutamate levels and the pressure on energy metabolism, Rao et al. (2009) found excitotoxicity and neuroinflammation in post-mortem frontal cortex from BD patients.

Studies using transcranial magnetic stimulation paradigms showed a significant deficit in cortical inhibition in BD patients compared to healthy controls (Levinson et al., 2007; Chroni et al., 2008), which is in agreement with the data showing increased glutamatergic neurotransmission in BD patients. The hippocampus, another brain region affected in BD, also is the site of adult neurogenesis (Vadodaria and Gage, 2014). New excitatory granule cells are continuously generated in the dentate gyrus. After maturation and integration in the neural circuit, the new neurons are indistinguishable from those generated during embryonic development, but it is their hyperexcitable nature during maturation that gives the hippocampus its plasticity and particular cognitive functions (Kempermann et al., 2015). Studies using several mouse models of psychiatric disorders, such as the Ca^2+^/calmodulin-dependent protein kinase II (α-CaMKII) heterozygous knockout, showed that the dentate gyrus granule cells were arrested at a stage with similar molecular and physiological properties to those of the immature neurons (Yamasaki et al., 2008; Hagihara et al., 2013). This phenotype was named “immature dentate gyrus” and was proposed to be an endophenotype of BD and other psychiatric disorders (Hagihara et al., 2013).

## Models of BD and Clinical Translation to Drug Target

The development of novel treatments for psychiatric disorders has been hindered by the slow progress in our understanding of the underlying neurobiology, which results from the difficulty of developing faithful animal and cellular models. The complexity of psychiatric disorders and the still unknown relationships between the diagnosis and the etiology, neurobiology, genetics or response to medication led to the endophenotype concept. Endophenotypes are simple measurable heritable components that can be neurophysiological, biochemical, endocrine, neuroanatomical, cognitive or neuropsychological (Gould and Gottesman, 2006). Endophenotypes are useful in the construction of animal models and help dissect genetics and biological mechanisms of specific features of the disorders. Using this strategy, numerous rodent models of BD depression and mania have been constructed using approaches as diverse as genetic, pharmacological, nutritional and environmental (Nestler and Hyman, 2010; Kato et al., 2016; Logan and McClung, 2016). The use of animal models of human disease in research and drug testing should meet three criteria: construct validity, face validity and predictive validity (Nestler and Hyman, 2010). These criteria are useful in the evaluation of how similar is the animal model to the human disease in terms of shared genetics and mechanisms (construct validity), symptoms (face validity) and efficiency of medications on the animal phenotypes (predictive validity). Mice with a loss of function mutation in the CLOCK gene (*ClockΔ19* mutant mice) exhibit mania symptoms similar to those observed in patients, including hyperactivity, decreased sleep, lowered depression-like behavior, lower anxiety and an increase in the reward value for cocaine, sucrose, and medial forebrain bundle stimulation (Roybal et al., 2007). Interestingly, these symptoms were also reversed by chronic lithium administration. However, there is no evidence for circadian gene mutations in the majority of BD patients. To date, none of the BD models have fulfilled the requirements needed for their use in drug development, but they contributed to the understanding of the pathophysiology of the disorder (Nestler and Hyman, 2010; Kato et al., 2016; Logan and McClung, 2016).

Transgenic mice with overexpression of glycogen synthase kinase-3β (GSK-3β) show hyperactivity as observed in the manic phase of BD (Prickaerts et al., 2006). Lithium inhibits GSK-3β and this effect has been suggested as one possible mechanism of action in BD patients (Stambolic et al., 1996; Li et al., 2010). Lithium and valproate also act on the GSK-3β signaling pathway to reverse the manic-like behavior in an animal model of mania induced by ouabain (Valvassori et al., 2016). Ouabain inhibits Na^+^/K^+^-ATPase activity, which induces hyperactivity. Both lithium and GSK-3β knockdown act on circadian rhythm by producing a lengthening of *mPer2* period in mouse fibroblasts (Kaladchibachi et al., 2007). In addition, GSK-3β also phosphorylates PER2 (Iitaka et al., 2005) and REV-ERBα (Yin et al., 2006) regulating localization and stability of these proteins.

Lithium has numerous molecular targets, such as inositol monophosphatase (Agam et al., 2002; Harwood, 2005), protein kinase C pathway (Newberg et al., 2008) and calcium channels (Andreazza and Young, 2014) just to name a few. It is still an open question as to which one is responsible for the anti-manic effect in humans (Shaldubina et al., 2001; Beaulieu et al., 2008); nevertheless, these molecular pathways are used as targets to develop novel drugs or repurpose old ones. Long-term treatment of rats with lithium carbonate decreased membrane associated PKC in hippocampal structures (Manji et al., 1993) and treatment with valproate sodium increased cytosol/membrane ratio of PKC activity (Chen et al., 1994). Tamoxifen, a centrally acting PKC inhibitor has been shown to demonstrate anti-manic properties (Yildiz et al., 2008) in a randomized control trials of humans. More recently, the antioxidant ebselen, which inhibits inositol monophosphatase and induces lithium-like effects on mouse behavior, was suggested as a safe alternative to lithium (Singh et al., 2013). In anterior cingulate region of the brain, ebselen was shown to reduce glutamate and inositol levels possibly by inhibiting glutaminase (Masaki et al., 2016).

The advent of cellular reprogramming technology has allowed for the generation of iPSCs from somatic tissues (e.g., skin and blood) from patients with neuropsychiatric disorders (Takahashi et al., 2007; Brennand et al., 2011; Mertens et al., 2015). Significant methodological advances in human iPSC differentiation protocols has enabled iPSC-disease modeling using specific neuronal subtypes (Maroof et al., 2013; Nicholas et al., 2013; Zhang et al., 2013; Yu et al., 2014; Vadodaria et al., 2016a,b).

Chen et al. (2014) reported differences in transcriptional profiles in iPSC-derived neurons from controls and BD patients. The expression of transcripts for membrane-bound receptors and ion channels was significantly increased in BD neurons (Chen et al., 2014). Control neurons expressed transcripts that confer dorsal telencephalic fate, whereas BD neurons expressed transcripts that are involved in the differentiation of ventral regions (e.g., medial ganglionic eminence). iPSC technology allows for the interrogation of cellular phenotypes that can be detected during neuronal development and are not directly evident in post-mortem studies. Specifically for BD, neurodevelopmental deficits have been suggested (O’Shea and McInnis, 2016), but the lack of access to embryonic tissues has hindered the confirmation of a neurodevelopmental hypothesis. Evidence for altered neuronal development in BD has also been suggested using iPSC-derived neural progenitor cells (NPCs) from BD patients (Madison et al., 2015). In this study, the authors derived and characterized a set of 12 iPSC lines from a quartet of two BD-affected brothers and their two unaffected parents and found significant differences in neurogenesis and in expression of genes involved in WNT signaling and ion channel subunits (Madison et al., 2015). Subsequent treatment of the NPCs with a pharmacological inhibitor of GSK-3β (CHIR99021), a known regulator of WNT signaling, rescued the progenitor proliferation deficit in the BD patient NPCs. The role of micro RNAs (miRNAs) was also investigated in BD neuronal tissue and cultures. Bavamian et al. (2015) showed increased levels of miR-34a in post-mortem cerebellar tissue from BD patients, as well as in BD patient iPSC-derived neuronal cultures. miR-34a is predicted to target multiple genes implicated as genetic risk factors for BD, and the authors have validated a number of predicted mir-34a direct targets in the BD cultured neurons (ankyrin-3, ANK3 and voltage-dependent L-type calcium channel subunit beta-3, CACNB3; Hunsberger et al., 2013; Bavamian et al., 2015). In addition, overexpression of miR-34a was shown to result in abnormalities in neuronal differentiation and morphology as well as in the expression of synaptic proteins in control cells (Bavamian et al., 2015). The authors propose that miR-34a regulates a molecular network essential for neuronal development and synaptogenesis that is implicated in BD neuropathology.

A study examined hippocampal DG granule cell neurons (Prox1 positive) differentiated from six BD patients and four healthy controls (Mertens et al., 2015). Gene expression studies were performed and suggested mitochondrial abnormalities in DG granule cell neurons from BD patients. Interestingly, electrophysiological functional studies revealed hyperexcitability in BD neurons that was selectively decreased after lithium treatment in neurons from lithium-responsive patients, and not in neurons from the non-responders (Mertens et al., 2015). This work suggests that clinical information and drug response patterns can be used to test the validity of cellular phenotypes in culture. That realization is very powerful since it could open new avenues to find new drugs and therapies that ameliorate phenotypes in cultured neurons and could potentially be translated into patient treatments.

## Conclusion and Future Challenges

The conventional drug development approach for psychotropics has been through the manipulation of receptor profiles of existing drugs or purely by an empirical approach. Neuroscience-based treatment development for psychiatric disorders has stagnated over the last four decades, with molecular and neuroscience research findings often not mapping onto clinical phenomenological approaches. One of the reasons for the slow progress is the lack of BD accurate animal and cellular models for drug testing and pathophysiology investigation. The expansion of studies using iPSC-derived technologies hopefully will allow for a better understanding of the affected molecular pathways and provide an initial platform for drug development. Despite the fact that it seems impossible to have animal models of psychiatric illness that fully reproduce the complex neurologic symptoms and associated comorbidities, animal testing for new drugs before clinical trials is an obligatory step. The construction of valid animal disease models is thus a foremost challenge.

The integration of other symptoms observed in BD patients, besides the neuropsychiatric, and medical comorbidities led to the exploration of essential cellular functions, not specific to neurons but shared by multiple cell types. The early observation of altered brain energetic metabolism encouraged the search for mitochondrial dysfunction. Mitochondria are central organelles in a cell and even minor dysfunction can lead to a cascade of changes and damage. To ensure survival, the cells adapt to chronic mitochondrial dysfunction coordinating responses with other organelles, such as the ER. However, survival of BD cells has a cost on physiology and ultimately causes perturbations in different tissues and organs. In addition, BD is a neurodevelopmental disorder and mitochondrial metabolism modifications are essential during neurogenesis (Zheng et al., 2016). Following this line of thinking, drugs that target these pathways are potentially interesting for BD treatment, as primary or adjuvant medicine. For example the use of minocycline, which is an antibiotic that can modulate glutamate-induced excitotoxicity and has antioxidant and anti-inflammatory properties, showed promising results in clinical trials (Dean et al., 2012). We believe that a better understanding of the molecular mechanisms that result in mitochondrial impairment and oxidative stress together with the regulation of adaptive UPR and autophagy responses will provide the key pieces of information that will unlock novel drug treatments for BD beyond mood stabilizers and antipsychotics.

## Author Contributions

YK, RS and MCM designed and wrote the article. FHG contributed with discussions and revision of the text.

## Conflict of Interest Statement

The authors declare that the research was conducted in the absence of any commercial or financial relationships that could be construed as a potential conflict of interest.
